# Loose programming of GIS workflows with geo‐analytical concepts

**DOI:** 10.1111/tgis.12692

**Published:** 2020-10-26

**Authors:** Johannes F. Kruiger, Vedran Kasalica, Rogier Meerlo, Anna‐Lena Lamprecht, Enkhbold Nyamsuren, Simon Scheider

**Affiliations:** ^1^ Department of Human Geography and Spatial Planning Utrecht University Utrecht the Netherlands; ^2^ Department of Information and Computing Sciences Utrecht University Utrecht the Netherlands

**Keywords:** automated workflow synthesis, core concepts of spatial information, geocomputation, geospatial semantic web

## Abstract

Loose programming enables analysts to program with concepts instead of procedural code. Data transformations are left underspecified, leaving out procedural details and exploiting knowledge about the applicability of functions to data types. To synthesize workflows of high quality for a geo‐analytical task, the semantic type system needs to reflect knowledge of geographic information systems (GIS) at a level that is deep enough to capture geo‐analytical concepts and intentions, yet shallow enough to generalize over GIS implementations. Recently, core concepts of spatial information and related geo‐analytical concepts were proposed as a way to add the required abstraction level to current geodata models. The core concept data types (CCD) ontology is a semantic type system that can be used to constrain GIS functions for workflow synthesis. However, to date, it is unknown what gain in precision and workflow quality can be expected. In this article we synthesize workflows by annotating GIS tools with these types, specifying a range of common analytical tasks taken from an urban livability scenario. We measure the quality of automatically synthesized workflows against a benchmark generated from common data types. Results show that CCD concepts significantly improve the precision of workflow synthesis.

## 1 INTRODUCTION

**FIGURE 1:**
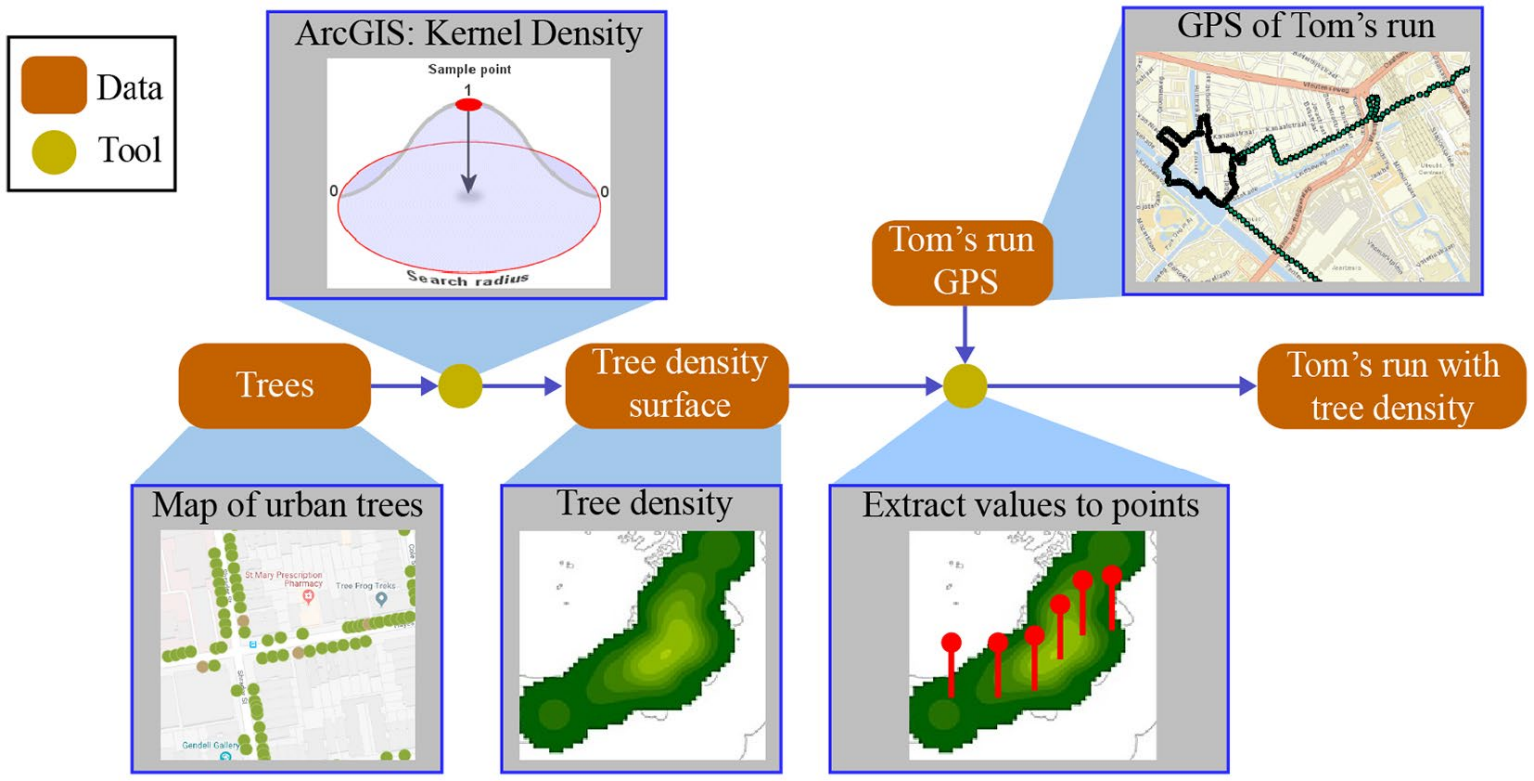
Example of a geo‐analytical workflow in GIS

Creating useful workflows for analysis with geographic information systems (GIS) requires a lot of expertise. This includes background knowledge of data sources, about their semantics, formats and data qualities, knowledge of GIS functions, as well as a sound understanding of the analytic goal to be pursued. For example, in the context of health geography, suppose our task is to assess how much a person (say, Tom) is exposed to health‐relevant environmental factors when running through the city of Amsterdam (Figure 1). Different kinds of GIS tools (e.g., kernel density, distance, and spatial interpolation) might be applied to different kinds of input data (e.g., an inventory of trees as points, maps of land use, and pointwise measures of air quality) to design a valid workflow for assessing the exposure of the run. Producing such workflows is difficult and hard to automate, given the level of expertise and the growing amount of tools and data sources to be familiar with (Scheider, Nyamsuren, Kruiger, and Xu, 2020).

An essential scientific challenge for geographic information science is therefore to understand what kind of knowledge is needed to automate this task.

In the past, researchers have suggested semantics‐based geoservice descriptions and service matchings to help analysts achieve a computational goal (see Section 2; see also Hofer, Mäs, Brauner, & Bernard, 2017). However, this approach had limited success for several reasons. For one, it remained unclear *at what level GIS data* should best be described (Hofer, Mäs, et al., 2017). The state‐of‐the‐art data types, such as vector and raster, do not capture important analytic constraints. For instance, in our example above, the *point vector data* remains ambiguous without human interpretation as to whether it can be used for density estimation or rather for spatial interpolation. Furthermore, since different tool sets capture similar *functionality at different levels of granularity*, it seems hard to design a general tool ontology (see Albrecht, 1998). For example, while the different *spatial point interpolation* operations, such as inverse distance weighting (IDW) and Kriging, appear as separate functions in ArcGIS (https://pro.arcgis.com/en/pro‐app/tool‐reference/3d‐analyst/an‐overview‐of‐the‐raster‐interpolation‐toolset.htm), they are merely parameters of a single function in R (https://rspatial.org/raster/analysis/4‐interpolation.html). Finally, geo‐operator thesauri and pairwise service matching (Brauner, 2015) may help retrieve tools for a given purpose. Yet, generating a workflow involves creativity in synthesizing an answer, which goes beyond information retrieval. For example, in the task above, a different yet equally valid approach might involve analyzing land use patches instead of trees. That means the actual *synthesis process* of GIS workflows itself needs to be taken into focus. Unfortunately, this has hardly been done in previous works (see Section 2).

*Loose programming* is an emerging software engineering paradigm that focuses on automatically synthesizing programs based on loose specifications (Lamprecht, Naujokat, Margaria, & Steffen, 2010). In this approach, instead of explicitly calling functions in procedural code, a user feeds *computational goals and other constraints* into an algorithm, which generates workflows of a maximal length satisfying these constraints. Constraints are expressed in terms of *semantic concepts* over data inputs, desired outputs, and functions. The algorithm reasons over concept hierarchies and function signatures to compose adequate workflows. This approach has so far only sporadically been applied to geographic information processing (Kasalica & Lamprecht, 2019).

To exploit loose programming for GIS, the quality and usefulness of workflows as well as the computational complexity depend largely on the quality and depth of semantic models used to describe GIS functionality. If semantic models are too shallow, they do not capture important distinctions necessary for applying geospatial analysis tools (Scheider & Tomko, 2016). Returning to our example, this means that a spatial data type, such as *point vector data*, is not sufficient for knowing that a GIS tool, such as IDW, is meaningfully applicable. Rather, we need to know that, at a more conceptual level, points represent measurements of spatially continuous phenomena, such as temperature, and hence depict a field. This in turn means that we need to be able to distinguish point data sets that represent *fields* from those that represent *discrete objects* (Scheider, Gräler, Pebesma, & Stasch, 2016). On the other hand, if the semantic model is too specific, it misses functional equivalences, such as the one between Kriging‐ and IDW‐based spatial interpolation, or between equivalent tools in different software environments. A basis for building such a semantic model are *core concepts of spatial information*, which were suggested by Kuhn (2012) as a generic interface to GIS, functioning as conceptual lenses an analyst may use to interpret spatial features. The core concepts were recently formalized into the *core concept data type (CCD) ontology*, which is a semantic data type system which captures the different ways in which core concepts can be represented in geodata at different levels of measurement (Scheider, Meerlo, Kasalica, and Lamprecht 2020). CCD types allow us to add necessary geo‐analytical constraints and capture functionality in terms of transformations in four semantic dimensions.

However, an empirical study of the expected *quality gain* of loose programming for GIS workflow synthesis using CCD types is missing. For this purpose, we suggest an *evaluation method*, measuring GIS workflow synthesis quality along different dimensions. Furthermore, we propose a *benchmark* of the capacity of GIS functionality descriptions using currently available data types. Such benchmarking also provides a novel way of measuring the impact of geospatial semantics (Janowicz, Van Harmelen, Hendler, & Hitzler, 2014) on the quality of analytic products, thus highlighting the relevance of semantics for data analysis (Scheider, Ostermann, & Adams, 2017). Based on this, we test a way to automatically synthesize GIS workflows by loosely specifying computational goals, input data sources, and tools in terms of abstract geo‐analytical concepts. We test concepts at the level of core concepts, geometric layer types, extensive attributes, as well as measurement levels, using five different geo‐analytic scenarios. For each scenario, the quality of workflows generated by specifying GIS tool signatures and queries in the full semantic model is measured against workflows built from a “shallow” semantic model in the benchmark. Results show that the precision of the suggested workflow set is raised significantly and that the CCD ontology enables GIS workflow composition at a high level of quality.

We start with a discussion of previous work on GIS workflow composition and process synthesis (Section 2), before reviewing geo‐analytical concepts in the CCD ontology, used to specify computational signatures of ArcGIS tools (Section 3). We then introduce our framework for loose specification of geo‐analytical tasks (Section 4) and for workflow synthesis evaluation (Section 5). Evaluation results are discussed in Section 6.

## 2 WORKFLOW SYNTHESIS AND GEOSERVICE COMPOSITION

In this section we review preliminary work on the synthesis of workflows, the retrieval of workflow resources and geoservice composition in the geospatial domain.

### 2.1 Geoservice composition and geo‐ontologies

The general idea behind automated workflow composition is to provide a framework that can automatically translate an abstract task description into an executable workflow. In practice, these approaches strongly depend on the availability of rich tool annotations and controlled vocabularies formalizing domain‐specific constraints. Automated program and workflow composition is a challenging and active field of research in computer science (Gulwani, Polozov, & Singh, 2017), but it has not been intensively studied in the geospatial domain so far. Though tool ontologies (Albrecht, 1998) and abstract GIS operations (Chrisman, 2002, Section 2, pp. 103–242) have been known for decades, they do not seem to have matured to the stage of automated workflow composition. Still, we can distinguish a few different approaches with the aim of simplifying the creation of GIS workflows. Most of them agree that an information ontology is a suitable formalism for structuring existing data types and operations (Athanasis, Kalabokidis, Vaitis, & Soulakellis, 2009; Hofer, Mäs, et al., 2017; Lemmens et al., 2006; Visser, Stuckenschmidt, Schuster, & Vogele, 2002; Yue, Baumann, Bugbee, & Jiang, 2015; Yue, Di, Yang, Yu, & Zhao, 2007). This is justified by the fact that different tasks may require different levels of constraints and explanations (Uschold & Jasper, 1999), both being provided by an ontology. The existing approaches can be classified according to their preferential focus on the workflow synthesis process.

Some authors provide an intuitive interface for helping users discover GIS tools and data sources for workflow composition (Athanasis et al., 2009; de Jesus, Walker, Grant, & Groom, 2012; Lemmens et al., 2006; Wiegand & García, 2007). Müller (2015) recently proposed hierarchical profiles for service discovery. These approaches still rely on manual workflow composition, similar to workflow management approaches (see Hull et al., 2006) such as Visser et al. (2002) and Ludäscher et al. (2006). Here, the focus is on using formal semantics to simplify the transition process between data sources. The same holds for workflow repositories based on linked data (Scheider & Ballatore, 2018). Some authors have proposed *task‐centered ontologies* for service chaining and data retrieval (Wiegand & García, 2007), and use it to retrieve and invoke workflows from a knowledge base (Sun, Yue, Lu, Zhai, & Hu, 2012; Zhuang, Xie, Ma, Guo, & Wu, 2018).

Other authors (Athanasis et al., 2009; Hofer, Mäs, et al., 2017; Yue et al., 2007) aim to automate the process of *GIS workflow composition* itself. Most of these authors focus on the semantic discovery of individual operations from a knowledge base, based on either formal input and output specifications (Athanasis et al., 2009; Fitzner, Hoffmann, & Klien, 2011; Lutz, 2007), or tool thesauri (Brauner, 2015; Hofer, Mäs, et al., 2017; Hofer, Papadakis, & Mä, 2017). Although operation discovery is a crucial step in workflow discovery, there is still a need to combine the discovered operations in executable workflows. Yue et al. (2007) address the type chaining problem and provide automated discovery of chains of operations, based on their input/output specifications. Farnaghi and Mansourian (2013) used a planning algorithm to automatically find solutions to the sheltering problem in disaster management. These latter approaches are comparable to the technical problem we address here. Yet, from an ontological viewpoint, they seem to lack a crucial distinction between semantic (conceptual) and syntactic (format) data properties (Kuhn, 2012; Kuhn & Ballatore, 2015). This distinction is seldom drawn, yet we believe it is required to capture how concepts can be represented by different geodata formats. In a nutshell, our idea is that, whenever analysts compose workflows, they interpret data in a way that *adds missing semantic information* to make effective use of the data (Scheider, Meerlo, et al., 2020). Furthermore, task specifications in the form of explicit application constraints (e.g., “perform an operation of type X”) are not supported, and systematic validations of workflows are still lacking.

In conclusion, although all of these approaches address workflow discovery, none of them supports fully automated workflow synthesis. For this purpose, APE (Automated Pipeline Explorer; Kasalica & Lamprecht, 2020a, 2020b) was recently introduced as a system that implements fully automated workflow discovery based on previous work on scientific workflow synthesis (Lamprecht, 2013). APE offers a lot of flexibility in reusing data and operation ontologies as sources for reasoning in the workflow construction process. One of the essential elements of effective workflow composition is a sufficient supply of *semantic constraints* captured in a formalized ontology. The field of GIS does not have a ready‐to‐use ontology for workflow composition (Hofer, Mäs, et al., 2017). Furthermore, map‐making and geo‐spatial analysis are full of implicit semantic intricacies, and therefore creating GIS workflows goes far beyond fitting geodata types to inputs and outputs (Scheider, Ballatore, & Lemmens, 2019).

### 2.2 Loose programming and workflow synthesis

The workflow composition approach that we focus on in this article accommodates user interaction at an abstract level, including abstract goals and functional constraints, by exploiting function and data hierarchies. The whole process consists of three phases:

1. In the *domain modeling* phase, lightweight ontologies (in the form of semantic hierarchies) written in the Web Ontology Language (OWL, https://www.w3.org/TR/owl2‐overview/) are provided that classify data and operations, together with tool annotations, that is, descriptions of the input and output types of the tools.
2. In the *task specification* phase, a specification is given of raw data as input and data that is required as an output of the process. Also, diverse logical constraints are specified over the workflow, such as “never use tool X” and “if tool *X* is used, tool *Y* cannot be used subsequently”.
3. The final phase is *workflow synthesis*, which is completely automated. It involves reasoning over the given workflow specification and ontology sources and finally providing a set of workflows that satisfy the given specification.

Both the domain modeling and the last two phases may be repeated until a desired solution is discovered. The idea is that the domain model is provided by a small group of experts and is utilized by a larger group of users in phases 2 and 3. The implementation of this approach in APE was inspired by the PROPHETS loose programming framework (Lamprecht et al., 2010; Naujokat, Lamprecht, & Steffen, 2012) and uses the same underlying semantic linear‐time logic (SLTL) synthesis method (Steffen, Margaria, & Freitag, 1993). The approach has already successfully been used in different bioinformatics (Lamprecht, 2013; Palmblad, Lamprecht, Ison, & Schwämmle, 2018) and geoinformatics (Al‐Areqi, Lamprecht, & Margaria, 2016; Kasalica & Lamprecht, 2019; Scheider, Meerlo, et al., 2020) applications. For example, in Kasalica and Lamprecht (2019) we used APE to synthesize cartographic workflows for the automatic creation of maps depicting bird movement patterns in the Netherlands. We used tools from the GMT (Generic Mapping Tools; Wessel, Smith, Scharroo, Luis, & Wobbe, 2013) collection as the basis for the workflow composition. For another example, in Scheider, Meerlo, et al. (2020) we used the CCD ontology to compute a livability atlas of Amsterdam using standard GIS operations as provided by ArcGIS. While all these applications were very promising, an empirical study of the workflow synthesis quality has yet to be done.

## 3 GEO‐ANALYTICAL CONCEPTS AND GIS TYPE SYSTEM

In this section we summarize our previous work on the CCD ontology, which provides the semantic basis for this workflow construction study. The lightweight ontology CCD was introduced in Scheider, Meerlo, et al. (2020) and is formalized in the Web Ontology Language (OWL).^1^ CCD defines data types as intersections of OWL classes representing combinations of geo‐analytical concepts from four semantic dimensions: (a) *geometric layer types*, which generalize geometric properties of layers; (b) *core concepts* of spatial information (Kuhn, 2012), which capture what these layers represent; (c) *measurement levels* of attributes; as well as the notion of (d) *extensiveness*. These four dimensions are considered largely independent of each other, which makes them separable sub‐ontologies. The summary below follows the discussion in Scheider, Meerlo, et al. (2020). For details, we refer to the original sources. The ontology is available online (http://geographicknowledge.de/vocab/CoreConceptData.rdf) and all resources and annotation files used in this study can be found on GitHub (https://github.com/simonscheider/SemanticPipelines/tree/cf3c5af3a0114cf502fceee1e0578127e2e8cdf2).

### 3.1 Geometric types of layers

A GIS *layer* is a monothematic foil over the Earth’s surface. It is a data set with a type of spatial geometry common to all its data items as well as a spatial extent. The geometrical properties of a layer make it more or less suitable to represent geo‐analytic concepts within its extent. Every data record can have multiple attributes, one of them being a geometry. For example, a PointData record contains a single point geometry with multiple other attributes.

Layers can be of the types listed in Table 1. We distinguish not only raster from vector and point, line, or region layers, but also generalizations such as tessellations, which are mutually non‐overlapping and jointly covering sets of spatial regions. They can be both raster or vector. Tessellations are suitable for representing certain core concepts, namely “fields,” because they can specify a value for every point inside their spatial extent. However, as we will see in the following, other semantic interpretations are possible. We retain the classical distinction between raster and vector even though it is not essential for the representation of concepts (Scheider, Meerlo, et al., 2020), simply because it is a technical constraint for tool applicability, and thus helps us synthesize workflows.

**TABLE 1:** Data types of GIS layers

Data type definition/axiom	Explanation
*SpatialDataSet*≡∀*hasElement*.*SpatialData*	GIS layer (with elements that have spatial data geometries)
*PointDataSet*≡∀*hasElement*.*PointData*	Layer with only point geometries
*LineDataSet*≡∀*hasElement*.*LineData*	Layer with only line geometries
*RegionDataSet*≡∀*hasElement*.*RegionData*	Layer with only region geometries
*Tessellation*⊏*RegionDataSet*	Layer where the regions are space‐filling and non‐overlapping
*Raster*⊏*Tessellation*	A raster layer (tessellation where the regions are equal‐sized squares)
*Vector*≡*SpatialDataSet* ⊓ ­*Raster*	A GIS vector layer
*VectorTessellation*≡*Vector* ⊓ *Tessellation*	A vector tessellation

### 3.2 Core concepts and core concept data types

The core concepts of spatial information^2^ (2012) are cognitive lenses through which the environment can be regarded. In the CCD ontology, we consider them as concepts that are represented by a given layer (Scheider, Meerlo, et al., 2020). A distinction is made between a base concept (*location*), two quality concepts (*granularity* and *accuracy*), and four content concepts (*field*, *object*, *event*, and *network*). In this article we focus on the following two content concepts:

1. *Field*. A spatial(‐temporal) field describes a spatial(‐temporal) phenomenon that is defined everywhere inside some extent in space (and time). It can be thought of as a function with a domain that is a metric space, and a range that is a quality of some phenomenon. Examples include air temperature, land use, distance to nearest hospital.
2. *Object*. An object is an individual with an identity and spatial qualities such as boundaries that can change in time. An object can also have other qualities. Examples include houses, trees, cities.

The reason for our focus is, apart from limitations of space, that the concepts listed dominate standard GIS applications. *Spatial network* and *event* (including the temporal dimension) not only require special functionality, but also arguably evolved later in the evolution of GIS (Geertman, de Jong, & Wessels, 2003; Siabato, Claramunt, Ilarri, & Manso‐Callejo, 2018), and are thus considered future work.

Since core concepts have the properties mentioned, certain kinds of operations are naturally applied to them. For example, since fields are functions on a metric space, the quality of the phenomenon that they describe can be probed at any distance within their extent. This is not the case for objects. On the other hand, objects can be counted, have spatial parts (mereology) and neighbors (topology), and give rise to sizes and closeness. In essence, we exploit the idea that when analysts use GIS on a geodata source, they implicitly interpret not only their analytical goal, but also the data source and GIS tools in a way that is best captured by these core concepts.

Core concepts can be represented by GIS layers in different ways. In principle, core concepts and layer types can mix independently; however, certain combinations occur more often, and thus are captured as type combinations in the CCD ontology, while others are deprecated. For example, objects are usually not represented by rasters, at least not in a direct manner, since objects are seldom squared (Scheider, Meerlo, et al., 2020). As illustrated in Table 2, CCD defines classes as intersections of core concepts (Section 3.2) and geometry data types (Section 3.1). For example, *ObjectDS* ⊓ *PointDataSet* is the class of GIS layers with points that represent objects. An example would be a layer of trees. Alternatively, spatial objects may also be represented by a tessellation, as in the case of administrative units, which is then called a *LatticeDS*. A tessellated *field* representation, in contrast, is called a *CoverageDS*. Here, the regions do not denote boundaries of discrete objects, but rather enclose homogeneous values of continuous fields. An example would be a layer of patches of types of land use, where each patch is interpreted as a region of homogeneous land use values. Similarly, a *CountourDS* represents a field in terms of contour regions, each corresponding to a value interval, as in the case of noise or height contours. Finally, a field can also be represented by point‐like measures (*PointMeasuresDS*), as in the case of temperature measurements, by a raster layer (*FieldRasterDS*), as in the case of remote sensing images, or by arbitrary vector geometries. Note that core concept type combinations in this way add meaning to a given layer type.

**TABLE 2:** CCD definitions and axioms

CCD definition/axiom	Explanation	Example
*ObjectDS*⊏*SpatialDataSet*	Layer that represents objects	Trees
*LatticeDS*≡*ObjectDS* ⊓ *Tessellation*	Layer that represents objects that happen to be tesselated	Neighborhoods of Amsterdam
*FieldDS*⊏*SpatialDataSet*	Layer that represents a field	Noise levels
*CoverageDS*≡*FieldDS* ⊓ *Tessellation*	Layer that represents a field by giving values of the field for regions in the Tessellation	Land use
*ContourDS*⊏*CoverageDS*	Coverage where the regions are indicated to be in specific intervals of some ordinal scale	Noise levels
*PointMeasuresDS*≡*FieldDS* ⊓ *PointDataSet*	Point layer with measurements of a field	Temperature measurements
*FieldRasterDS*≡*FieldDS* ⊓ *Raster*	Raster layer that represents a field	

### 3.3 Measurement level and extensiveness of attributes

Another useful geo‐analytical distinction at the level of layer attributes is provided by measurement levels. Originally developed by Stevens (1946), they were further developed by Chrisman (2002, Chapter 1, pp. 15–35), imposing restrictions on the kinds of operations that can be performed on values of a given level of measurements (Chrisman, 2002, Section 2, pp. 103–242). In GIS, measurement levels can express, for example, that Kriging interpolation can only be ‐performed on attributes that are at an interval level, and that forming attribute ratios results in a ratio level. We denote the corresponding concepts at the attribute level with an “A” suffix; for example, *NominalA* denotes attributes at a nominal scale level (Table 3). Note also that our measurement levels include more than the standard ones (e.g., a count scale).

**TABLE 3:** Attribute type definitions and axioms

Attribute definition/axiom	Explanation	Example
*Attribute*	Any attribute of a layer.	Population (of a layer with cities)
*NominalA*⊏*Attribute*	Attribute on a nominal scale (can be compared for identity)	Land use type
*BooleanA*⊏*NominalA*	Attribute on a nominal scale with only two possible values	Is policy in effect here?
*OrdinalA*⊏*NominalA*	Attribute on an ordinal scale (can be compared for less than/equal to as well)	Disease intensity of a tree
*IntervalA*⊏*OrdinalA*	Attribute on an interval scale (a difference is meaningful)	Temperature (in degrees Celsius)
*RatioA*⊏*IntervalA*	Attribute on an ratio scale (ratios and 0 are meaningful)	Temperature (in kelvin)
*CountA*⊏*RatioA*	Attribute that represents a count of discrete entities	Number of sport facilities
*ERA*⊏*IntervalA*	Attribute that represents an extensive region attribute	Gross domestic income
*IRA*⊏*IntervalA*	Attribute that represents an intensive region attribute	Population density

Furthermore, we also make use of a distinction between *extensive* and *intensive* region attributes, as proposed in Scheider and Huisjes (2018). Extensive attribute values are dependent on the size of their region and behave in an additive manner, such that the sum of the attribute values of two regions equals the attribute value of the sum of their regions (e.g., population counts). Intensive attributes are independent of this size (e.g. area normalized attributes, such as population density). This semantic distinction has a direct impact on the applicability of spatial aggregation methods or areal interpolation.

### 3.4 Combining semantic dimensions

Core concept data types combine *geometric layer types* and *core concepts* at the level of data sets, as well as *measurement levels* and *extensiveness* on the level of geodata attributes. Formally, these combinations were defined at the level of attributes, by introducing corresponding attribute types for all types of layers mentioned above, by simply adding “A” as a suffix. For example, an attribute of a tessellation (*TessellationA*) might occur at an ordinal measurement level (*OrdinalA*).

**FIGURE 2:**
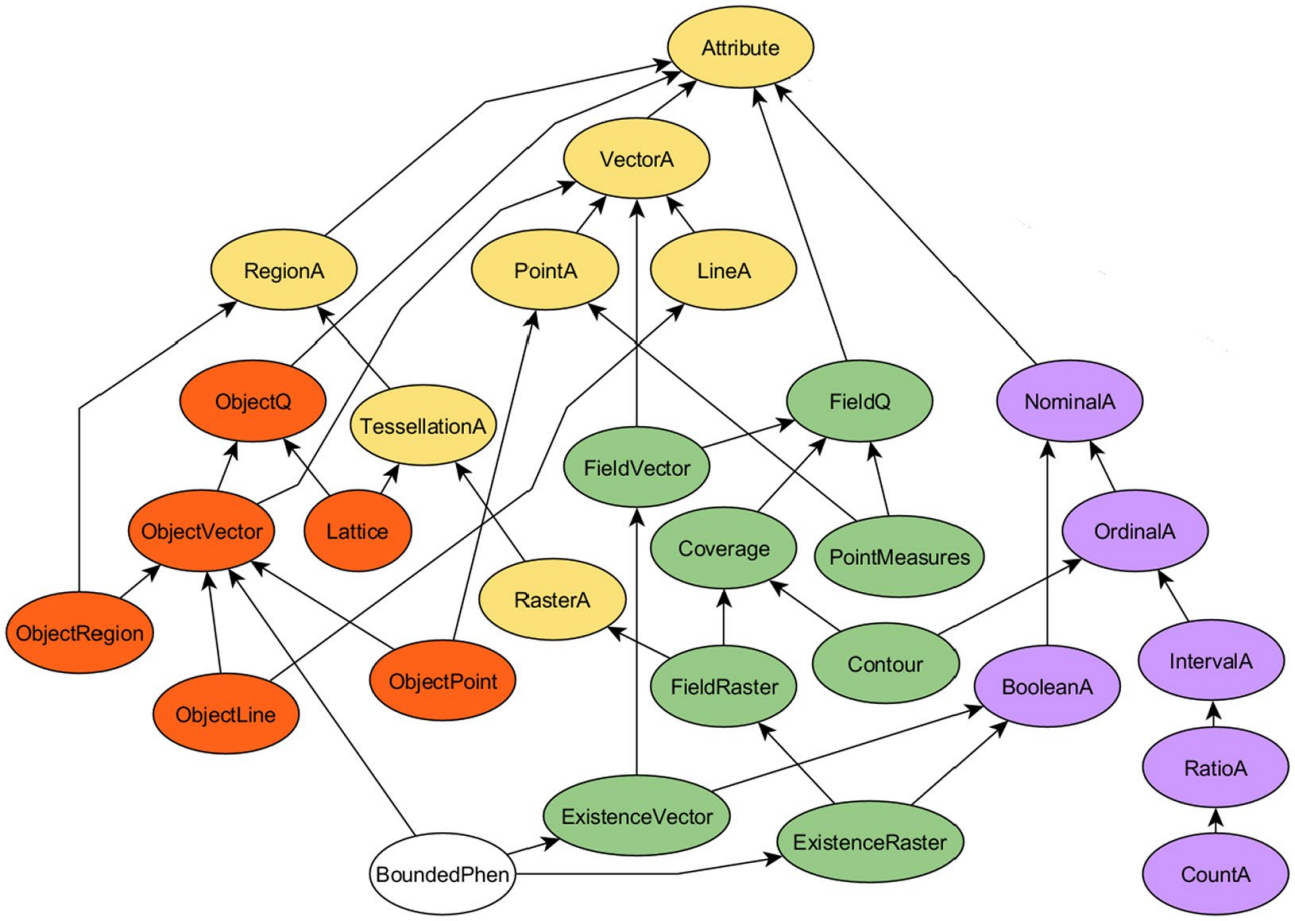
Overview of the different semantic dimensions of the CCD ontology and some frequent class combinations at the attribute level. Red denotes object attributes, green field attributes. Measurement levels are denoted by violet classes, and geometric attribute types by yellow ones

The CCD ontology includes frequent kinds of combinations. For example, a tessellation data set which represents a field in terms of nominal attribute values was defined as a *Coverage* (Figure 2). Each semantic dimension corresponds to a subsumption hierarchy of classes (Figure 2, implied by colors and the roots *ObjectQ*, *FieldQ*, *NominalA*). Since the workflow synthesizer (APE) searches for subsumed classes only within a single data type hierarchy and assumes a single class for each data input and output, we need to add all occurring class combinations. To reduce search complexity, we also required each tool to be annotated only at the most specific (leaf) level, assuming that these leaf classes are mutually exclusive. We therefore synthesized missing leaf nodes for all occurring combinations in a programmatic manner.
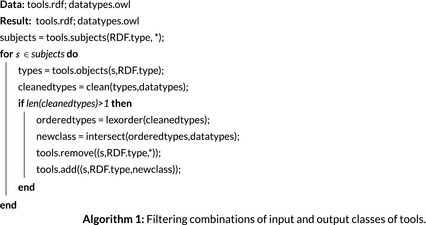


Such intersections are added based on Algorithm 1 (https://github.com/simonscheider/SemanticPipelines/blob/cf3c5af3a0114cf502fceee1e0578127e2e8cdf2/typeCombinations.py). We assume here that tools are annotated in an RDF file (*tools.rdf*), such that a tool’s inputs and outputs are modeled as blank nodes instantiating potentially many classes defined in an OWL‐based datatype ontology (*datatypes.owl*). The latter contains several independent hierarchies, one for each semantic dimension. We used rdflib‐based queries (https://github.com/RDFLib/rdflib/) over subjects and objects of triples. In Algorithm 1, we iterate over all subjects of class instantiations in *tools.rdf*, corresponding to some data input or output. Then we filter and clean the list of types by removing supertypes. If more than one type remains, we order them lexicographically and then intersect them using OWL class intersections. This is done by first checking whether the ontology already contains the intersection class, and if not, adding corresponding OWL triples to the ontology. Subsumption relations of the intersected classes are then inferred based on OWL reasoning. In this article we used OWL2‐RL reasoning (https://www.w3.org/TR/owl2‐profiles/#OWL_2_RL) to this end.

An alternative to this approach would be to deal with class combinations along separable semantic dimensions directly in the workflow synthesis algorithm. This would make the approach independent of other reasoners and simplify concept search. Such dimensional reasoning is ongoing work within the further development of APE.

### 3.5 Geocomputational signatures

The types of the CCD ontology are used for formulating input and output constraints of tools. This is also called an operational *signature*. The tools that we use to compose workflows come from ArcGIS Pro (https://www.esri.com/en‐us/arcgis/products/arcgis‐pro), one of the major GIS software environments. Note, however, that this can be easily exchanged with a different GIS such as QGIS (https://qgis.org). For the selection of tools that we annotated, we specified core concepts, geometric layer types, and measurement levels of the inputs and outputs of the tools. Take the example of *map algebra* (Tomlin, 2013), a type of operation that applies algebraic expressions over coincident cell values across different raster layers. In terms of core concepts, *local map algebra* operates on fields, and in terms of layer types, it operates on rasters. This means it receives two FieldRasters as input, and results in a FieldRaster as well. In contrast to local map algebra, *zonal map algebra* results in a Lattice, and thus can be used for aggregation. The FieldRasters used as input to map algebra can be at various measurement levels, depending on the specific kind of algebraic expression given as a parameter to the method. For example, a *local product* operation requires at least ratio scale. Wherever it made a difference, we additionally specified extensiveness of interval scaled attributes, as in the case of a *focal sum* operator. A focal sum operator sums cell values in a defined neighborhood around each cell in the input raster, and this means that values need to be extensive counts, such as ”number of species”. An excerpt of the geocomputational signatures is listed in Table 4. The full *tool annotation file* is available online (https://github.com/simonscheider/SemanticPipelines/blob/cf3c5af3a0114cf502fceee1e0578127e2e8cdf2/ToolDescription.ttl), and a more extended explanation of the annotation process can be found in Scheider, Meerlo, et al. (2020).

**TABLE 4:** Geocomputational signatures of GIS tools (excerpt)

GIS function	Input type	Second input	Output type
Local map algebra (prod)	*FieldRaster* ⊓ *RatioA*	*FieldRaster* ⊓ *RatioA*	*FieldRaster* ⊓ *RatioA*
Focal statistics (mean)	*FieldRaster* ⊓ *IntervalA*		*FieldRaster* ⊓ *IntervalA*
Focal statistics (sum)	*FieldRaster* ⊓ *CountA* ⊓ *ERA*		*FieldRaster* ⊓ *CountA* ⊓ *ERA*
Focal statistics (density)	*ExistenceRaster*		*FieldRaster* ⊓ *RatioA*
Zonal statistics (median)	*FieldRaster* ⊓ *OrdinalA*	*Lattice* ⊓ *VectorA*	*Lattice* ⊓ *VectorA* ⊓ *OrdinalA*
Boolean reclassify	*FieldRaster*		*ExistenceRaster*
Spatial join (count)	*ObjectVector*	*Lattice* ⊓ *VectorA*	*Lattice* ⊓ *VectorA* ⊓ *CountA*
Spatial join (mean)	*ObjectVector* ⊓ *IntervalA*	*Lattice* ⊓ *VectorA*	*Lattice* ⊓ *VectorA* ⊓ *IntervalA*
Kriging/IDW	*PointMeasures* ⊓ *IntervalA*		*FieldRaster* ⊓ *IntervalA*
ThiessenPolygons	*PointMeasures* ⊓ *NominalA*		*Coverage* ⊓ *NominalA*
Polygon to raster	*Coverage* ⊓ *NominalA*		*FieldRaster* ⊓ *NominalA*
Raster to polygon	*FieldRaster* ⊓ *NominalA*		*Coverage* ⊓ *NominalA*
Raster to contour	*FieldRaster* ⊓ *IntervalA*		*Contour* ⊓ *OrdinalA*
Raster to polyline	*ExistenceRaster*		*ObjectLine* ⊓ *BooleanA*
Extract values to points	*FieldRaster* ⊓ *NominalA*	*PointMeasures*	*PointMeasures* ⊓ *NominalA*
Select by attribute	*Coverage*		*ExistenceVector*
Euclidean distance	*ObjectVector*		*FieldRaster* ⊓ *RatioA* ⊓ *IRA*
Kernel density	*ObjectVector*		*FieldRaster* ⊓ *RatioA* ⊓ *IRA*
Feature to raster	*ObjectVector*		*ExistenceRaster*
Areal interpolation (mean)*	*Lattice* ⊓ *VectorA* ⊓ *IntervalA* ⊓ *IRA*	*Lattice* ⊓ *VectorA*	*Lattice* ⊓ *VectorA* ⊓ *IntervalA* ⊓ *IRA*
Merge features	*ExistenceVector*		*ObjectVector*

*Notes*: Tool parametrization is given in parentheses. An asterisk * means that methods are actually a pipeline of different tools that are used together. See tool annotation file for further details.

To capture parametric variations of operations and to preserve their inherent semantics at the most specific level, we frequently had to *overload* tool annotations. For example, the *focal statistics* tool can be used with many different operational parameters (mean, sum, density), each with a different signature. Also, the “mean” variant might have inputs at various measurement levels (e.g., interval, ratio, count), enforcing outputs at corresponding levels. For each of these possibilities, we added a distinct subtool with a different type signature and a slightly different name (such as *FocalStatisticsMeanInterval*; see tool annotation file). The resulting hierarchy of tools encodes different versions of focal map algebra.

## 4 LOOSE SPECIFICATION OF GEO‐ANALYTICAL TASKS

To apply and test our framework for loose programming of GIS workflows, we use a scenario with typical geo‐analytical questions that can be handled by a GIS. The scenario revolves around livability in Amsterdam, and it uses openly available (https://data.amsterdam.nl) data from the city of Amsterdam and comparable sources. The general task is to derive livability indicators for elderly people for each postcode area at level 4 (PC4) in Amsterdam, using different urban environmental factors which make the area livable for the elderly. Coping with the diversity of these factors makes the scenario challenging. For this study, we formulated five questions as detailed below.^3^

For each question, we give a short motivation and specify the geo‐analytical tasks that are used in the evaluation. Specifications are available online^4^ and are later used in the evaluation framework. Each task involves extracting goal concepts, choosing data sets for generating answers in terms of start concepts, and tool specifications were used whenever the question included hints at corresponding functions. Note that more sophisticated kinds of specifications might be used in the synthesis process (see Scheider, Meerlo, et al., 2020). Our specifications using the CCD ontology are listed in Table 5. The “BDT” version of these specifications in the same table corresponds to the benchmark data types ontology, a taxonomy of common GIS types (explained later in Section 5.1).

**FIGURE 3:**
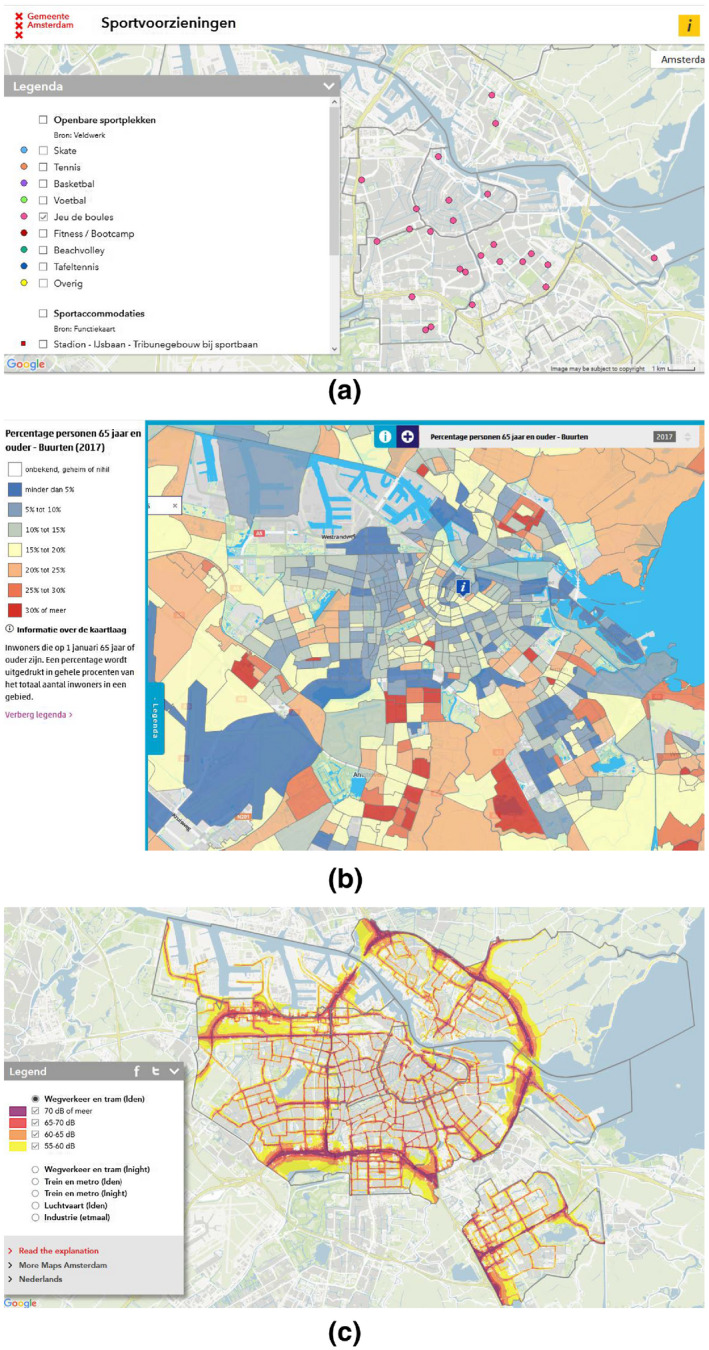
Map data sources to assess livability, taken from https://maps.amsterdam.nl/open_geodata/. (a) Sport facilities (Jeu de Boule) in Amsterdam. This helps answer question 1. (b) CBS Buurt statistics, showing the percentage of persons over 65 in neighborhoods. This answers question 2. (c) Noise map of the Amsterdam municipality, with intervals given in decibels. This can help answer question 4

1. *What is the number of sports facilities in each PC4 area? Motivation*: Elderly people might prefer particular facilities, such as places for playing pétanque or boules.
*Given data*: Sports facilities (Figure 3a) are interpreted as objects, and represented by point vectors with a nominal attribute denoting the facility type; PC4 areas^5^ in Amsterdam form a vector lattice.
*Goal specification*: The goal is a vector lattice at the PC4 level with extensive counts.
2. *What is the proportion of elderly people living in each PC4 area in Amsterdam? Motivation*: Elderly people may prefer neighborhoods where they can meet their peers, or may conversely be happy to live in an area with many young people.
*Given data*: The CBS Buurt statistics (Figure 3b) contain percentages of elderly in the population of a neighborhood. These are interpreted as a vector lattice with ratio scaled (intensive) attribute.
*Goal specification*: The goal is a ratio scaled intensive attribute of a vector lattice at the PC4 level.
3. *What is the accessibility of parks for each PC4 area in Amsterdam? Motivation*: Elderly people might prefer living in neighborhoods where parks are within reach so they can easily take a walk.
*Given data*: The CBS land use data set (https://www.cbs.nl/nl-nl/dossier/nederland-regionaal/geografische-data/natuur-en-milieu/bestand-bodemgebruik) can be used to select areas with parks. It is interpreted as a Coverage with nominal attribute denoting the land use type (“park en plantsoen”).
*Goal specification*: The goal is a ratio scaled attribute of a vector lattice at the PC4 level.
*Tool specification*: Also, the term “accessibility” in the question implies an answer which makes use of some distance measurement.
4. *What is the amount of noise pollution in each PC4 area in Amsterdam? Motivation*: Elderly people might prefer living in neighbourhoods where there is low noise.
*Given data*: The map of traffic noise levels (Figure 3c) is interpreted as a Contour map with an ordinal attribute denoting the noise interval in decibels.
*Goal specification*: The goal is an ordinal scaled attribute of a vector lattice at the PC4 level.
*Tool specification*: The term “amount” implies aggregating the noise field over the PC4 area. Therefore we added the constraint that some aggregation method, such as zonal aggregation, should be used.
5. *What is the average temperature within each PC4 area in Amsterdam? Motivation*: Elderly people are especially sensitive to urban heat islands, so they might prefer neighbourhoods with low average/maximum temperature in the summer.
*Given data*: A map of pointwise meteorological measurements^6^ with an interval scaled attribute denoting temperature.
*Goal specification*: The goal is an interval scaled attribute of a vector lattice at the PC4 level.
*Tool specification*: As above, the term ”average” implies aggregating the temperature field over the PC4 area. Therefore we added the constraint that some aggregation method, such as zonal aggregation, should be used.

**Table 5:** The questions and their input, goal and tool specifications in the CCD and the benchmark (BDT) ontology

Question	Ontology version	Input specification	Goal specification	Tool specification
What is the number of sport facilities in each PC4 area?	CCD	ObjectPoint⊓ NominalA	CountA⊓VectorA⊓Lattice⊓ERA	
		Lattice⊓ VectorA		
	BDT	PointA	VectorRegionA	
		VectorRegionA		
What is the proportion of elderly people living in each PC4 area?	CCD	Lattice⊓ VectorA⊓ RatioA⊓ IRA	Lattice⊓VectorA⊓RatioA⊓IRA	
		Lattice⊓VectorA		
	BDT	VectorRegionA	VectorRegionA	
		VectorRegionA		
What is the accessibility of parks for each PC4 area in Amsterdam?	CCD	Coverage⊓ NominalA	RatioA⊓VectorA⊓Lattice	Distance
		Lattice⊓ VectorA		
	BDT	VectorRegionA	VectorRegionA	Distance
		VectorRegionA		
What is the amount of noise pollution in each PC4 area in Amsterdam?	CCD	Contour	OrdinalA⊓VectorA⊓Lattice	ZonalStatistics
		Lattice⊓VectorA		
	BDT	VectorRegionA	VectorRegionA	ZonalStatistics
		VectorRegionA		
What was the average temperature within each PC4 area?	CCD	IntervalA⊓ PointMeasures	IntervalA⊓VectorA⊓Lattice	ZonalStatistics
		Lattice⊓VectorA		
	BDT	PointA	VectorRegionA	ZonalStatistics
		VectorRegionA		

As one can see in these examples, our loose specifications exploit the information given in the questions as well as the information about available data sources to the largest possible extent. This includes specific semantic interpretations of the sources. Though such interpretations might be done differently in some cases (Scheider, Meerlo, et al., 2020), we believe the chosen ones represent a defendable expert view of the analytic tasks. Note that, though their specifics are given, solving these analytic tasks still involves non‐trivial expert knowledge. For example, based on reading question 5, a layman might believe one could simply “average” the given pointwise temperature measurements, while the task actually requires estimating and summarizing a field. The former would result in a semantic error, rendering the workflow meaningless and therefore useless for the purpose. It is interesting to test whether CCD can add this level of expert knowledge into the synthesis process.

The design and testing of our ontology and workflow composition system went through two development stages. In a *preliminary test*, we compiled a comparable but slightly different set of questions, including *What is the distance to the nearest voting office for the area of Amsterdam?*, *What is the average distance to the nearest sports facility within each PC4 area?*, and *What is the most common land use type in each PC4 area?* instead of questions 2, 3 and 4. The preliminary test deviated also in other respects.^7^

## 5 EVALUATION FRAMEWORK

In general, workflows can be evaluated at *design‐time* or *run‐time*. Whether a workflow is actually executable can only be evaluated at run‐time and involves automatic deployment. However, even if a workflow is readily executable, it still might generate meaningless results that do not answer the question. For this reason, we are more interested in assessing the *meaningfulness* of an answer (Scheider et al., 2016), and this can already be done at design‐time using expert assessments. This section explains our framework for doing this, and the results are discussed in Section 6.

**FIGURE 4:**
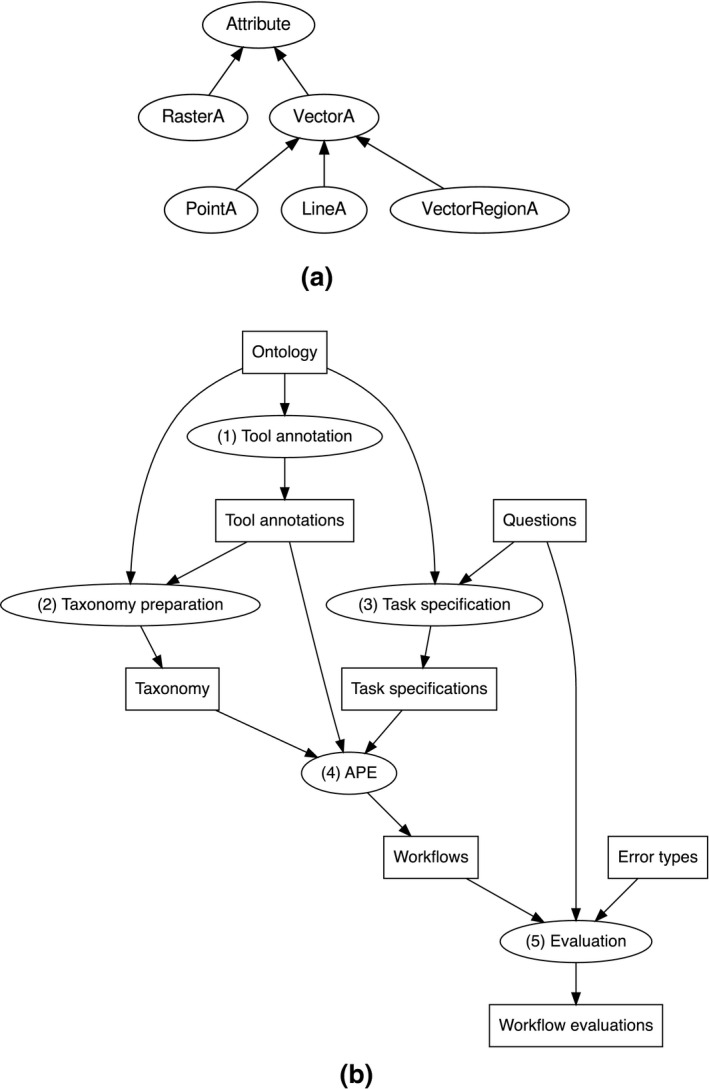
Elements of our evaluation framework. (a) The BDT ontology only consists of the well‐known raster and vector data types, and a further specification of the different vector types. It is a subset of the CCD ontology defined at the attribute level. (b) A summary of our ontology evaluation framework for workflow synthesis. For an ontology, five steps are performed (see text). All steps are done both for the CCD ontology and the benchmark ontology as a benchmark to measure improvements

The evaluation of an ontology for workflow synthesis consists of multiple steps (Figure 4b). (a) The tools are annotated with data classes from the ontology (signatures; see Section 3.5). For example, the fact that Kriging interpolation transforms a PointMeasure data set into a FieldRaster data set is annotated here. (b) In a taxonomy preparation phase, the ontology and the annotated tools are used to create a *taxonomy* of types. This taxonomy is an RDF schema (RDFS) hierarchy (consisting of *rdfs:subClassOf* triples) of data and tool classes. Note that this preparation step also includes OWL reasoning in order to infer such statements for the defined class combinations occurring in the tool annotation, and a removal of other types of triples (see Section 3.4). (c) Analytical questions are coded into task specifications, consisting of goal, input and tool specifications (see Section 4). (d) The taxonomy, tool annotations, and task specifications are fed into APE, which generates a set of up to *n* distinct workflow solutions of length *k* for each specification. After first experimentations, *n* was set to 20 and *k* to 8, because it became clear that longer and larger numbers of workflows only increased redundancy (see Section 6). (e) The quality of the solutions is evaluated by a GIS expert using the error classification scheme explained in Section 5.2 and a solid understanding of the questions. The evaluation process including APE configuration and output workflow graphs is documented online,^8^ and the taxonomy preparation is documented here (https://github.com/simonscheider/SemanticPipelines/tree/cf3c5af3a0114cf502fceee1e0578127e2e8cdf2).

### 5.1 Workflow synthesis benchmarking

In order to benchmark the CCD ontology, we compared the synthesized workflows against workflows obtained under the exact same conditions, except that we used a different type system. The reason for doing this is to be able to measure the improvement that conceptual/semantic types add to workflow synthesis. The benchmark should reflect the types provided by current geodata structures. More precisely, we generated a proper subset of CCD where all conceptual dimensions (including core concepts and measurement levels) were removed and which only includes one semantic dimension related to geometry types, namely the distinction between raster and vector attributes, as well as between point, line and region attributes (Figure 4a). Note that the class Tessellation was also removed, since it does not occur in current data structures. We call this ontology the *benchmark data types* (BDT) ontology. Using this simple ontology, we manually created corresponding tool annotations (Section 3.5) by substituting every type with the least upper bound (supremum) concept that is still in BDT. In the same way, we generated BDT versions of the task specifications, as listed in Table 5.

### 5.2 Error types and precision measures

How can GIS workflows best be evaluated? We decided to use a quality assessment approach from information retrieval (Blair, 1979). The idea is that workflow synthesis is treated like a retrieval process, and its precision is measured by the extent to which the synthesized workflows answer the given question. In principle, one could measure both precision (the proportion of retrieved answers that are correct given all retrieved answers) as well as recall (the proportion of retrieved answers that are correct given all correct answers), however, the latter is difficult since it requires a complete and correct answer set generated by experts. Another problem is the definition of correctness in terms of error types. The quality of GIS workflows is evaluated using a schema of four error types at two different severity levels, explained below and summarized and illustrated in Table 6.

**TABLE 6:** An overview of the different error types

Error severity	Error type	Example workflows
Hard	Signature	Figure 5c
	Semantic imprecision	Figures 5a and b
Soft	Redundancy	Figures 6b, 7b and c
	Data quality	Figure 6c

*Hard errors* are critical errors which result either in a wrong or non‐meaningful answer, or in a workflow that is non‐executable due to wrong data formats. Correspondingly, we distinguish two kinds of hard errors: *signature* errors, which have a part of the workflow that cannot be executed because a tool is incorrectly applied; and *semantic imprecision* errors, which produce a meaningless or invalid answer for the given question, because the ontology misses some required semantic constraint of applicability of data, tools or some information contained in the question.

*Soft errors* are non‐critical errors where workflows *do* entail a correct answer, but which are in some sense of lesser quality. We distinguish two kinds of soft errors: *redundancy* errors, where workflows make use of tool applications which are unnecessary for giving a valid answer; and *data quality* errors, where workflows contain transformations that diminish the *geodata quality* of the result in a way that is unnecessary, but that still render the workflow useful for the task. Geodata quality has many dimensions, such as positional and attribute accuracy, granularity/precision (≈ resolution) and completeness (Guptill & Morrison, 2013). Geodata quality comes in degrees, so geodata is never perfectly accurate, precise, and complete. Furthermore, data transformations never increase the quality and GIS workflows usually entail some quality loss. In our case, quality errors mostly included unnecessary reductions of the *spatial resolution* (e.g., based on applying unnecessary focal statistics which tends to blur a raster). For example, the workflow in Figure 6c shows how an interpolated raster is blurred in this way before being aggregated with zonal statistics. We will discuss more examples of soft errors below.

**FIGURE 5:**
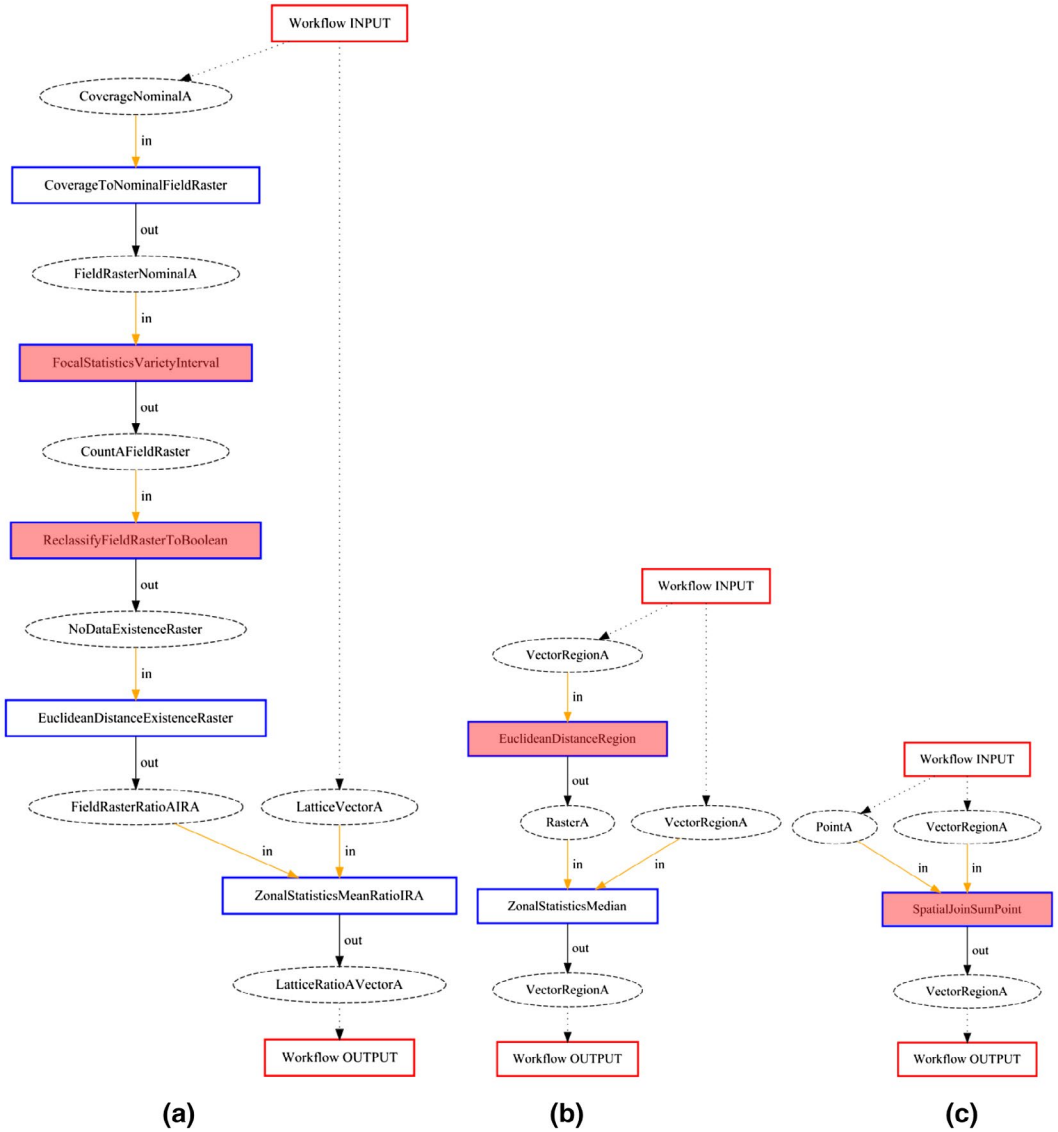
Examples of hard errors for workflows synthesized for questions 3 and 1. Erroneous function applications are highlighted in red: (a) Semantic imprecision error in workflow for question 3 (CCD); (b) Semantic imprecision error in workflow for question 3 (BDT); and (c) Signature error in workflow for question 1 (BDT).

Figure 5 illustrates three hard errors. For all of these, either the answer is not meaningful for the given question, or the workflow is not even executable. The workflow in Figure 5a is‘ supposed to answer question 3 about the accessibility of parks, and it was generated based on CCD. It converts land use polygons to a land use raster, and subsequently counts the variety of land use types in a neighborhood around each raster cell. This “land use diversity” is subsequently reclassified to an existence raster (e.g., by selecting a certain range; this is unspecified in our tools). The next operation calculates the Euclidean distance to this filtered land use diversity. Finally, the average distance to the filtered land use diversity is computed in each PC4 area. Clearly, this workflow is not meaningful for our question, and it is hard to imagine a scenario where it would be. For this reason, it is classified as a semantic imprecision error.

The workflow in Figure 5b is also supposed to answer question 3 and was generated based on BDT. It uses the land use data set *directly* for distance measurement, and therefore the resulting raster represents the distance to *any* land use polygon. Because the land use polygons cover the entire extent, this will always be 0, and is therefore not meaningful, and thus classified as a semantic imprecision error.

A signature error occurs in Figure 5c, which is supposed to answer question 1 about the number of sports facilities. Here, the points with sports facilities, which have a nominal attribute that indicates the facility type, are *summed* in every PC4 area. Nominal attributes are usually encoded with strings, but numbers are expected. For this reason, the workflow is not executable, and it is classified as a signature error.

## 6 RESULTS

To assess the value of the CCD ontology relative to the benchmark, we evaluated synthesized workflows in the manner described in the last section. In this section we report the results and discuss their implications.

### 6.1 Workflow evaluation

We counted errors for workflows that were synthesized with both ontologies in the preliminary study (Table 7) and the main study (Table 8). In these tables we report soft errors only for those workflows that did not have any hard errors. Thus, the sum of hard and soft errors can be at most equal to the number of workflows. The “correct” column shows the number of workflows without any hard errors.

**Table 7:** Breakdown of the errors for the preliminary study

				Hard errors		Soft errors	
Question	Ontology	Workflows	Correct	Signature	Semantic imprecision	Redundancy	Data quality
1	BDT	20	6	0	14	5	0
1	CCD	20	19	0	1	17	1
2	BDT	20	5	0	15	2	2
2	CCD	20	11	1	9	9	7
3	BDT	20	1	0	19	0	0
3	CCD	20	3	0	17	0	2
4	BDT	20	5	0	15	4	0
4	CCD	20	17	3	0	16	0
5	BDT	20	1	0	19	0	0
5	CCD	20	18	2	0	15	1
1–5	BDT	100	**18** (18%)	0	82	11 (61%)	2 (11%)
1–5	CCD	100	**68** (68%)	6	27	57 (83%)	11 (16%)

**TABLE 8:** Breakdown of the errors for the main study with questions and ontologies, including tool constraints

				Hard errors		Soft errors	
Question	Ontology	Workflows	Correct	Signature	Semantic imprecision	Redundancy	Data quality
1	BDT	20	2	13	18	3	0
1	CCD	20	20	0	0	19	3
2	BDT	20	2	4	18	0	0
2	CCD	8	8	0	0	7	0
3	BDT	20	2	0	18	0	2
3	CCD	20	19	0	1	7	6
4	BDT	20	3	0	17	0	2
4	CCD	4	4	0	0	3	0
5	BDT	20	5	0	15	0	3
5	CCD	20	20	0	0	14	12
1–5	BDT	100	**14** (14%)	17	86	3 (21%)	7 (50%)
1–5	CCD	72	**71** (99%)	0	1	50 (70%)	21 (30%)

In the *preliminary study*, 20 workflows were synthesized for each of the five questions and for each of the two ontologies, resulting in 200 workflows in total. Note that this study was done with earlier versions of CCD and BDT as well as APE (see Section 4). We start with these results here because we think it is insightful to illustrate the potential for improvement of the ontology and the approach. Table 7 shows a breakdown of all questions,^9^ using the error classification scheme from Section 5.2. It can be seen that, while signature errors are very rare overall, the CCD ontology results in a significantly higher rate of correct workflows (from 18 to 68%). Question 3 (“average distance to sport facilities”) scores worst and is responsible for the majority of hard errors. This is due to missing tool specifications, which did not allow the information about distances in the question to be exploited for workflow construction. On the other hand, redundancy errors and data quality errors went up considerably in the CCD case. However, since an older APE version was used, workflow synthesis was more prone to both soft and hard errors.

Table 8 shows the evaluation results of the *main study*, including 172 workflows in total (https://github.com/quangis/gis_workflow_generation/tree/61452627c50bb89e73df8759088ac06ffb6ae033/evaluation). This is less than 200 due to more restrictive ontological constraints, which often prevented APE from reaching the maximum of 20 workflows. It can be seen that the improvements we made with respect to APE as well as the tool annotations, ontology and task specifications directly show in the result. The result is consistent with the preliminary result, although there is a further deepening of the quality gap between BDT and CCD. We can see that the hard error rate falls from 86% down to below 1% for CCD. This means that while only 14% of all BDT workflows were correct and answered the questions, almost 99% of all CCD workflows were meaningful answers to the questions posed. This gap can be directly attributed to the missing semantics in BDT. It is also interesting to see that BDT provoked 17% signature errors, because common geodata types do not include information about certain attribute value types that are important for syntax errors. The preliminary study still had signature errors in the CCD case (Table 7), which completely disappeared because of our improvements.

However, it is also apparent that redundancy errors in the main study remained very frequent for CCD, falling only slightly from 83 to 70% of all correct workflows. This is again consistent with the preliminary study.

We suggest that CCD causes more redundant workflows compared to BDT, precisely because it enforces more restrictive conditions on workflow synthesis. In consequence, the only way to produce longer workflows is to concatenate redundant tools. This explanation is also consistent with another observation, namely that the number of workflows CCD produced is often lower than the upper limit (20, see questions 2 and 4), showing that the space of possibilities of reaching the goal is very limited. In other words, lower amounts of hard errors and higher redundancy/limited workflow diversity turn out to be two sides of the same coin. This becomes more clear when looking at example workflows (see below).

Furthermore, it is interesting that the data quality error, though reduced by CCD, is still rather high in all cases, showing that a sufficient constraint on geodata quality is not captured by our ontology. This is not surprising since a data quality specific constraint was not included in the synthesis specifications. For example, though two workflows both may aggregate data, the one producing a data set of higher resolution might be preferable because positional uncertainty is reduced. Yet, such measures and corresponding constraints require a different approach and are considered future work (see next section).

**FIGURE 6:**
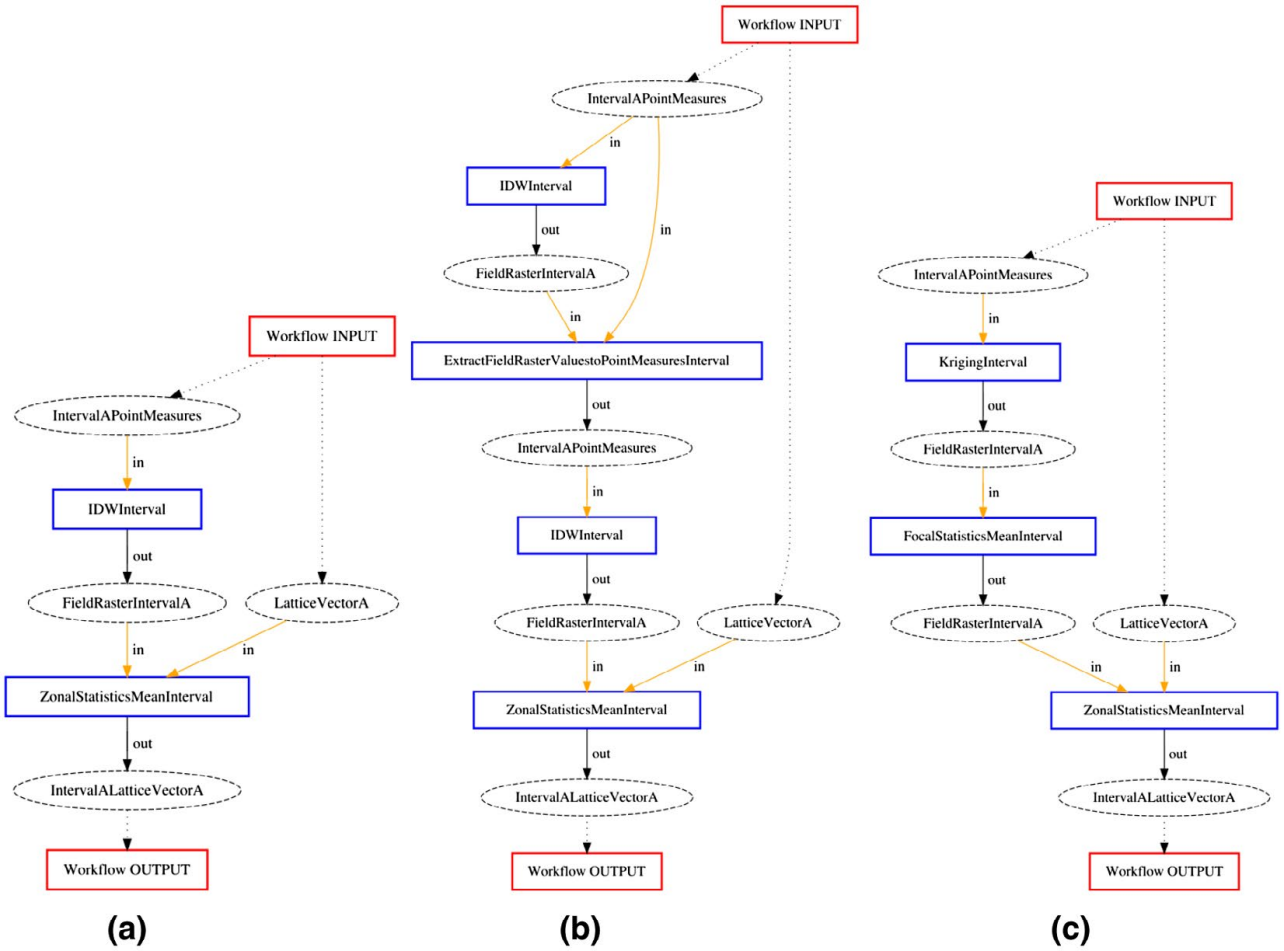
Examples of different soft error types for workflows synthesized for question 5 (“What is the average temperature within each PC4 area in Amsterdam?”) using the CCD ontology: (a) Correct and no soft errors; (b) Correct, but redundancy error; and (c) Correct, but data quality error

To better understand these results, we illustrate the workflows created by APE and their quality for question 5: “What is the average temperature within each PC4 area in Amsterdam?” Figure 6a shows a workflow which is a near perfect answer to the question. It takes the temperature measurements and performs inverse distance weighted interpolation to produce an interpolated temperature field raster. Subsequently, with zonal statistics, it uses the temperature field and the PC4 areas to compute the mean temperature in every PC4 area.

Figure 6b shows a different workflow with exactly the same result. The redundant part of this workflow starts when the temperature field raster is converted to temperature point measurements. The resulting IntervalAPointMeasures is (for all intents and purposes) *exactly* the same as the IntervalAPointMeasures that was provided as input. This is because the interpolated field is equal to the interpolated points’ values at the points’ locations, and exactly those locations are extracted from the field. After this redundant part, the workflow proceeds to calculate the correct answer as in Figure 6a.

**FIGURE A1:**
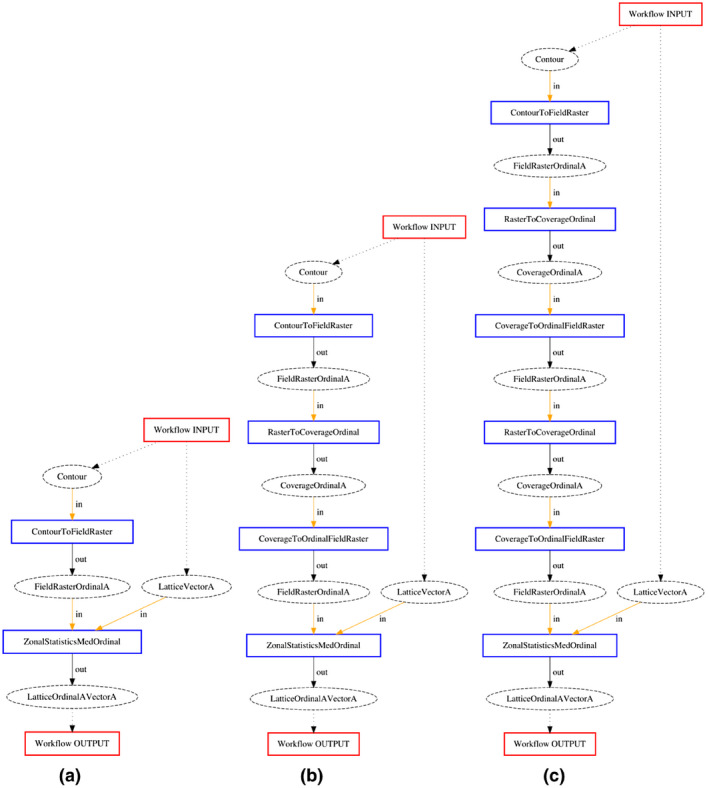
Examples of different soft error types for workflows synthesized for question 4 (“What is the amount of noise pollution in each PC4 area in Amsterdam?”) using the CCD ontology: (a) Correct and no soft errors; (b) Correct, but redundancy error; and (c) Correct, but data quality error

A more serious quality error occurs in Figure 6c. Here, the temperature field is blurred, because the application of FocalStatisticMeanInterval computes the mean of the temperatures within a radius of each raster cell. After this operation, the workflow calculates the answer in the same way as Figure 6a, but the resolution of the answer is decreased. Apart from these soft errors, concatenations and combinations of redundant and data quality errors also occur, and are also classified as soft errors. Further examples of workflows for question 4 can be found in the Appendix and Figure A1.

### 6.2 Discussion and future work

In a nutshell, our results demonstrate that loose programming with core concept data types as semantic constraints enables us to automate the design of GIS workflows for a diverse set of geo‐analytical tasks at a surprisingly high‐quality level. More precisely, this means that hard errors which would render the workflow useless for its intended purpose seem to be almost entirely prevented, given that appropriate input data of the right quality is available. This result has several important implications:

1. It indicates that common geo‐analytical *questions and tasks* might translate well to loose specifications using SLTL and the CCD ontology. Tasks including accessibility assessment, spatial interpolation and summary statistics can be specified using core concepts, measurement levels, as well as constraints over a semantic hierarchy of tool concepts.
2. It indicates that the CCD ontology might provide a solid *semantic basis for annotating GIS functions and data*, and for constraining their application to ones that are meaningful under the given task. This issue is not obvious, as it is still unknown which semantic level would be needed for geo‐analytical purposes. We have been able to demonstrate in this article that the benchmark geodata types are insufficient for this purpose and that the type system necessarily needs to go beyond this to capture the constraints implied by particular kinds of information concepts as well as measurement levels.
3. It indicates that our method of *benchmarking and evaluation* based on information retrieval might be used as a general method for quantifying the impact of semantic information on geo‐analytical task‐solving. Though semantic background knowledge is known to be important for data analytics (Scheider et al., 2017), it is commonly hard to measure its impact on information products. For this reason, ontology engineering often suffers from not being able to show its benefits. Workflow quality benchmarking provides a way to account for this.
4. It indicates that loose programming with CCD could be a way to approach the problem of indirect question‐answering (Scheider et al., 2017). In indirect question‐answering, questions cannot be answered directly, by retrieval or inference from knowledge bases, but they require adequate data transformations. GIS workflows are a very good example of the relevance of such a system, since geographic questions are seldom answerable without data transformations.

How might one make use of loose programming of GIS? While the vision of an entirely automated GIS still appears far fetched, we believe our study shows that semi‐automatic recommendations of geo‐analytical workflows for properly specified goals are within reach. In particular, they can support geoinformaticians and GIS analysts who develop workflows by systematically exploring the space of possibilities with the available tools. Though specifications still need to be formulated and answer workflows still need to be checked (and implemented) by human experts, our approach scales up the geo‐analytical process by automatically assessing the potential of a given tool resource for a task, which does not seem possible to date. It thus shows a way toward geocomputational code generation and question‐answering. A parallel opportunity is implied by the fact that CCD types describe geodata sources. Though the geodata retrieval problem admittedly involves more specific information about geographic phenomena than is captured by CCD, it might add to the effectiveness of current geographic information retrieval strategies (Jones & Purves, 2008).

These opportunities need to be seen in light of the inherent limitations of the current study. First, there are a number of technical improvements to be made. The run‐time deployment and evaluation of workflows is still future work. Also, the preparation of the CCD taxonomy currently has to deal with several semantic dimensions and arbitrary combinations thereof, while OWL reasoning is currently used to add missing subsumption relations. In the future, we plan to simplify this process by projecting annotations to a set of predefined semantic dimensions. This would allow APE to deal with these dimensions directly and more efficiently in the synthesis process. Furthermore, there remain a number of scientific challenges. For one, the translation of questions into CCD‐based SLTL specifications over workflows was done in a somewhat ad‐hoc manner. However, this is a challenge which warrants its own empirical research on semantic and syntactic structures of geo‐analytical questions using natural language processing interfaces and grammars. Secondly, scaling up the semantic annotation of data sources and diverse tool sets is a separate challenge which can be approached either by machine learning or crowdsourcing, both of which we are currently testing. And third, the evaluation of the data quality in workflow composition can be improved. For example, one could take into account data quality dimensions in the composition constraints. Both redundancy errors and data quality errors could be handled by exploiting restrictions in the sequencing of functions and by preferring shorter over longer workflows. A geodata quality measure would also add an additional dimension to core conceptual data type semantics, telling us something about a workflow’s degree of fitness for use in addition to its semantic appropriateness for a task.

Finally, another question concerns the *completeness* of the CCD ontology concepts concerning geo‐analytical tasks. Which concepts are we lacking and which are relevant for modeling some form of geospatial analysis? Are the current four semantic dimensions sufficient? This is probably not the case, as our workflow evaluation shows. For example, to capture certain functional constraints, such as “distance”, we need to be able to generalize over corresponding GIS tools. For this purpose, we are currently working on a *transformation language for geospatial information*. Furthermore, in the future, we plan to further develop and test the ontology on a more diverse set of geo‐analytic scenarios, including the core concepts event and network. Eventually, we expect to encounter a “law of diminishing returns,” which says that adding more semantic dimensions will raise the workflow quality but only to an lower and lower degree. This raises the question whether core concept data types together with the attribute types mentioned are the only way to reach the current level of workflow synthesis quality, or whether other semantic constraints could play the same role.

## 7 CONCLUSIONS

In this article we have investigated to what extent loose programming of GIS workflows with the CCD ontology is capable of automatically solving common geo‐analytical tasks. For this purpose, we annotated a GIS tool set with the ontology and evaluated the generated workflow sets according to their precision with respect to answering a set of five geo‐analytic questions given appropriate data sources. Precision was measured over 372 workflows, including a pre‐study, taking into account two hard and two soft error types. The performance of CCD was compared against a benchmark in geodata typing, which we believe can be regarded as a novel method of geo‐ontology benchmarking. Results show that the CCD ontology reduces the hard error rate from 86 to under 1%, but increases the soft error rate to 70%, in particular, the redundancy of workflows. This implies that the CCD ontology is effective in semantically constraining the synthesis process in a way useful for translating questions, determining data and tool sources, and for indirect question‐answering, yet it still lacks constraints related to geodata quality. The large soft error rate means that constraints are so tight that longer workflows tend to be simply more redundant. Furthermore, generalizable functional constraints are still lacking. In the future, we plan to do related research on question grammars, data and tool annotations and on improving the evaluation framework by better dealing with semantic dimensions. Furthermore, we plan to develop a data transformation language in a concerted effort to design a system for indirect geo‐analytical question‐answering.

## 8 ACKNOWLEDGEMENTS

This work has received funding from the European Research Council (ERC) under the under the European Union’s Horizon 2020 research and innovation programme (Grant agreement No. 803498).

## ENDNOTES

^1^ In the following we use the description logic notation for OWL (Krötzsch, Simancik, & Horrocks, 2012).

^2^ We will refer to these as the *core concepts* in the remainder of this article.

^3^ Note that assessing livability would in practice require more indicators, such as walkability, crowding and social security. For our purpose, however, the questions cover a set of sufficiently different concepts.

^4^ See configuration files at https://github.com/quangis/gis_workflow_generation/tree/61452627c50bb89e73df8759088ac06ffb6ae033.

^5^ PC4 areas are administrative regions sharing the first four digits of their postal code. They are always used as input data in the following and therefore are only mentioned once.

^6^ For example, as provided by the KNMI at http://www.klimaatscenarios.nl/toekomstig_weer/transformatie/index.html.

^7^ In the preliminary study (unpublished, see Meerlo, 2019), we used an earlier version of the ontology and the tool annotation, lacking some of the class combinations and including some incorrect annotations. Our task specifications were, in consequence, also slightly different. Furthermore, we did not use tool constraints, but only goal and data source specifications. We also used an older version of APE. See https://github.com/simonscheider/SemanticPipelines/tree/master/SatSolutionsCCDT.

^8^ See evaluation folder and APE configuration under https://github.com/quangis/gis_workflow_generation/tree/61452627c50bb89e73df8759088ac06ffb6ae033.

^9^ See the evaluation files of the study on figshare: https://doi.org/10.23644/uu.11791329.v1.

## APPENDIX A

Another remarkable collection of workflows is synthesized for question 4: “What is the amount of noise pollution in each PC4 area in Amsterdam?” Figure A1a is the correct workflow with no soft errors. It converts the noise contour to an ordinal raster, and computes the median noise level for every PC4 area. Figure A1b performs a redundant set of operations. The ordinal raster is converted to an ordinal coverage before being converted back into an ordinal raster. Then the workflow proceeds as it should. Figure A1c performs the redundant operations twice, and another workflow (not shown here) performs the redundant operations three times. It should be noted that all workflows using the CCD ontology for this question give the correct answer (albeit with soft errors in some cases), whereas the BDT ontology mostly produces meaningless results.
